# Attenuation of the Counter-Regulatory Glucose Response in CVLM C1 Neurons: A Possible Explanation for Anorexia of Aging

**DOI:** 10.3390/biom12030449

**Published:** 2022-03-14

**Authors:** Hajira Ramlan, Hanafi Ahmad Damanhuri

**Affiliations:** Department of Biochemistry, Faculty of Medicine, Universiti Kebangsaan Malaysia, Kuala Lumpur 56000, Malaysia; hajiraramlan@gmail.com

**Keywords:** anorexia of aging, caudal ventrolateral medulla, catecholaminergic neurons, glucoprivation, feeding response

## Abstract

This study aimed to determine the effect of age on CVLM C1 neuron glucoregulatory proteins in the feeding pathway. Male Sprague Dawley rats aged 3 months and 24 months old were divided into two subgroups: the treatment group with 2-deoxy-d-glucose (2DG) and the control group. Rat brains were dissected to obtain the CVLM region of the brainstem. Western blot was used to determine protein expression of tyrosine hydroxylase (TH), phosphorylated TH at Serine40 (pSer40TH), AMP-activated protein kinase (AMPK), phosphorylated AMPK (phospho AMPK), and neuropeptide Y Y5 receptors (NPY5R) in CVLM samples. Immunofluorescence was used to determine TH-, AMPK-, and NPY5R-like immunoreactivities among other brain coronal sections. Results obtained denote a decrease in basal TH phosphorylation levels and AMPK proteins and an increase in TH proteins among aged CVLM neurons. Increases in the basal immunoreactivity of TH+, AMPK+, NPY5R+, TH+/AMPK+, and TH+/NPY5R+ were also observed among old rats. Young treatment-group rats saw a decrease in TH phosphorylation and AMPK proteins following 2DG administration, while an increase in AMPK phosphorylation and a decrease in TH proteins were found among the old-treatment-group rats. These findings suggest the participation of CVLM C1 neurons in counter-regulatory responses among young and old rats. Altering protein changes in aged CVLM C1 neurons may attenuate responses to glucoprivation, thus explaining the decline in food intake among the elderly.

## 1. Introduction

Anorexia of aging refers to a decline in food intake and appetite attributed to advancing age. The phenomenon was first introduced by John Morley and Andrew J. Silver in 1988 [1]. The age-induced decrease in metabolic activity and alteration of appetite-regulatory hormones may be associated with anorexia of aging [2]. Low food intake in the elderly is also triggered by depression, asociality, and diseases [3,4]. Anorexia of aging could lead to undernutrition, frailty, sarcopenia, or even death [5,6].

Feeding behavior is regulated by interactions between different brain neurons and accompanying hormone signaling. The presence of ghrelin, cholecystokinin, and leptin in the feeding pathway has been well established. Catecholamines are also crucial mediators of the feeding pathway, given their role in promoting food intake and activating glucoregulatory responses [7,8]. At the cellular level, catecholamines and messengers such as AMP-activated protein kinase (AMPK), neuropeptide Y (NPY), and pre-opiomelanocortin are identified and often expressed in hypothalamic and brainstem neurons [9,10,11]. These regulatory hormones and proteins appear in the gut–brain axis, participating in and interacting with the regulation of energy homeostasis and food intake [7,12].

Caudal ventrolateral medulla (CVLM) C1 neurons are among the catecholaminergic neurons, which have been acknowledged as important in the production of glucose counter-regulatory responses. Previous studies utilized the glucose analogs 2-deoxy-D-glucose (2DG), 5-thio-D-glucose (5TG,) and insulin to stimulate hunger, create hypoglycemia, or induce glucoprivation to study glucoregulatory mechanisms [13,14]. 2DG increased activation of CVLM C1 neurons [13]. It also stimulated the expression of dopamine-beta hydroxylase (DBH) and NPY and increased the phosphorylation of tyrosine hydroxylase (TH) and AMPK [11,15].

According to John E. Morley, age-related changes in physiological activity are responsible for the anorexia of aging [2]. Many past studies established the involvement of CVLM C1 neurons in glucose regulation. However, evidence relevant to the effect of age on this mechanism remains limited. Therefore, by stimulating 2DG-induced glucoprivation, this study aimed to determine the effect of age on the function of CVLM C1 neurons in mediating the feeding response.

## 2. Material and Methods

### 2.1. Materials

Brain-tissue samples were collected using 2-deoxy-d-glucose, ethylene glycol tetra-acetic acid (EGTA), and reduced glutathione. Western blot and immunofluorescence analyses involved primary antibodies; primary monoclonal rat anti-tyrosine hydroxylase (T1299, Sigma-Aldrich, St. Louis, MI, USA), monoclonal rabbit anti-NPY5R (AB133757, Abcam, Cambridge, UK), polyclonal rabbit AMPKα1/2 (H-300) (sc-25792, Santa Cruz, CA, USA), polyclonal rabbit anti-phospho AMPKα (THR172, Millipore, Burlington, MA, USA), monoclonal mouse anti-β-actin (A5441, Sigma-Aldrich, St. Louis, MI, USA), and polyclonal rabbit anti-tyrosine hydroxylase phosphoSer40 (AB5935, Millipore, Burlington, MA, USA). Primary antibodies in Western blot analysis were detected using polyclonal goat anti-mouse IgG HRP (HAF007, R&D Systems, Minneapolis, MN, USA) and polyclonal goat anti-rabbit IgG (H + L) HRP (65-6120, Invitrogen, Waltham, MA, USA), whereas Alexa Fluor 488 conjugated with goat anti-rabbit IgG (H + L) secondary antibody (A11008, Thermo Fisher Scientific, Waltham, MA, USA) and CY3 conjugated with goat anti-mouse IgG (H + L) secondary antibody (A10521, Thermo Fisher Scientific, Waltham, MA, USA) were used in immunofluorescence.

### 2.2. Animals

Male Sprague Dawley rats aged 3 months and 24 months were used in this study. Animals were purchased from the Laboratory Animal Resources Unit (LARU), National University of Malaysia. All rats were housed in an animal cabin at suitable room temperature (20–25 °C) with ad libitum access to food pellets and water. The animal studies received ethical approval from the Universiti Kebangsaan Malaysia Animal Ethical Committee (UKMAEC) (approval number: FP/BLOK/2013/HANAFI/20-MARCH/499-MARCH-2012-FEB-2015).

### 2.3. Animal Treatment, Sample Collection, and Preparation

Saline (0.9% sodium chloride) and 2DG (400 mg/kg) were administered intraperitoneally (0.4 mL) to conscious animals (*n* = 6) before removal of food and water. At 30 min post-treatment, animals were anesthetized with anesthetics (i.e., combination of ketamine, xylazine, and zoletil-50; 0.4 mL/kg). Animals were decapitated upon non-response to painful stimuli. Whole brains were further sectioned using a coronal-plane brain matrix to obtain samples for Western blot (*n* = 6 per group). The caudal ventrolateral medulla (Bregma −13.0 to −14.0 mm) was identified based on a rat brain atlas [16] and stored at −80 °C until analysis. Animals transcardially perfused with 4% paraformaldehyde in 0.1 M phosphate-buffered saline (PBS, pH 7.4) had their whole brains rapidly removed and preserved overnight in the same fixative (*n* = 3 per group). The brains were then washed with PBS and cryoprotected using 30% sucrose in PBS at 4 °C until further analysis.

## 3. Immunofluorescence

Brainstems were mounted with Tissue-Tek OCT compound (Thermo Fisher Scientific, Waltham, MA, USA) in a cryostat (Thermo Fisher Scientific, Waltham, MA, USA) and sectioned on a coronal plane, producing 40 µM brainstem slices. All slices were stored free-floating in TPBS and then were washed three times for 10 min each time. They were then treated via the heat-induced antigen-retrieval method using saline–sodium citrate (SSC) buffer at 80 °C and shaken for 30 min. Next, blocking of tissue sections was performed with 10% normal horse serum (NHS) in TPBS for 1 h at room temperature. Tissue sections and primary antibodies (i.e., primary monoclonal rat anti-tyrosine hydroxylase (1:3000), monoclonal rabbit anti-NPY5R (1:1000), and polyclonal rabbit AMPKα1/2 (H-300) (1:1000) were then incubated overnight at 4 °C. Following washing (three times for 10 min each time) in TPBS, the sections were incubated in the dark with secondary antibodies (i.e., Alexa Fluor 488 conjugated with goat anti-rabbit IgG (H + L) secondary antibodies (1:1500) and CY3 conjugated with goat anti-mouse IgG (H + L) secondary antibodies (1:1000) containing 4% NHS in TPBS for 2 h at room temperature. The sections were washed again three times for 10 min each time. Upon final wash, the sections were mounted using DPX mounting medium and viewed under a fluorescence microscope (Olympus, Tokyo, Japan) using cellSens Standard software, version 1.14 [17]. The brain immunofluorescence sections were analysed according to Damanhuri et al. [13].

## 4. Western Blot Analysis

Homogenization of CVLM samples and protein determination were performed as in our previous study [18]. Next, polyacrylamide gel was loaded with 12 µg to 20 µg of protein per sample. A protein-blotting technique using a wet transfer system was then executed. TBST buffer (Tris, NaCl, and 0.1% Tween-20) was used to wash (three times for 10 min each time at moderate speed) the protein-containing membrane before it was incubated overnight at 4 °C with primary antibodies (i.e., primary monoclonal rat anti-tyrosine hydroxylase (1:600), monoclonal rabbit anti-NPY5R (1:40,000), polyclonal rabbit AMPKα1/2 (H-300) (1:1000), polyclonal rabbit anti-phospho AMPKα THR172 (1:80), monoclonal mouse anti-β-actin (1:2000), and polyclonal rabbit anti-tyrosine hydroxylase phosphoSer40 (1:800). Incubation of secondary antibodies (i.e., polyclonal goat anti-mouse IgG HRP (1:2000) and polyclonal goat anti-rabbit IgG (H + L) HRP (1:1000) was performed at room temperature for 2 h the following day. After the washing step, enhanced chemiluminescence (ECL) was added to the surface of the membrane to detect proteins. The membrane was viewed under a gel-documentation system. Representative blots are as shown in Figure S1, Supplementary Materials. Protein-band intensity was quantified using ImageQuant software.

## 5. Statistical Analysis

Statistical analyses were conducted via GraphPad Prism, version 7. All data were expressed as means ± SEMs. One-way analysis of variance (ANOVA) and Tukey’s post hoc test were employed to evaluate differences between experimental groups. Statistical significance was set at *p* < 0.05.

## 6. Results

### 6.1. Effect of Aging on TH, AMPK, and NPY5R Immunoreactivities in the CVLM

**Figure 1:**
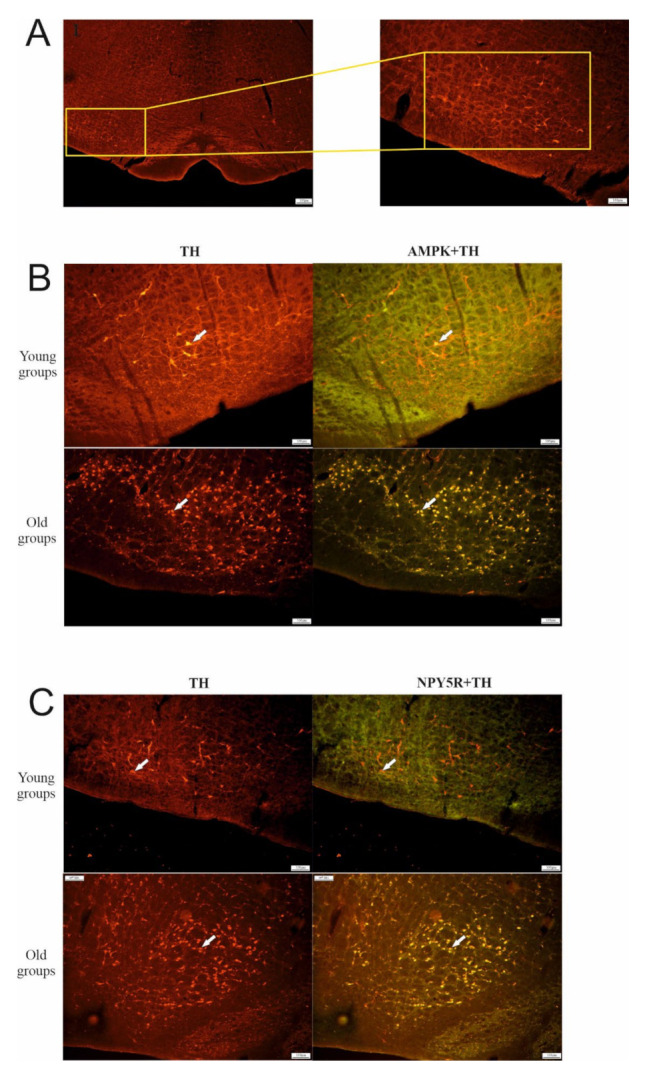
Distribution of TH-containing neurons and colocalization with AMPK or NPY5R in CVLM in different age groups. The highlighted area in the box indicates the CVLM region (**A**). TH co-localized with AMPK (**B**) and NPY5R (**C**) in CVLM neurons. The scale bars are 200 and 100 μm.

**Figure 2:**
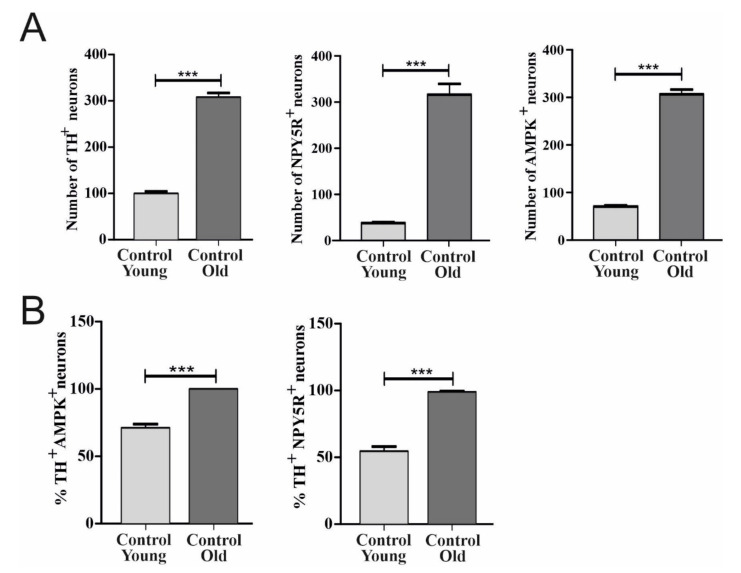
Effects of age on the distribution of TH, AMPK, and NPY5R in CVLM neurons. The numbers of neurons containing TH, AMPK, and NPY5R in CVLM were compared between age groups (**A**). Further analysis was conducted to show the colocalization of TH+/AMPK+ and TH+/NPY5R+ in the same brain region (**B**). Each bar and associated error bar represent the mean value of neuronal number ±SEM from one experiment (*n* = 3 per group). *** (*p* < 0.001) represents a significant difference between control groups of young rats and old rats, respectively.

Localization of TH, AMPK, and NPY5R in the CVLM and relevant age-related changes can be observed in Figure 1. In the CVLM, TH-, AMPK-, and NPY5R-containing neurons increased as age increased (*p* < 0.001) (Figure 2A). The expression of AMPK and NPY5R within TH-containing neurons was significantly higher in the old control group compared with the young control group (*p* < 0.001) (Figure 2B).

### 6.2. Effect of Aging and Glucoprivation on pSer40 TH, Total TH (tTH), Phosphorylated AMPK, Total AMPK (tAMPK), and NPY5R in the CVLM

**Figure 3:**
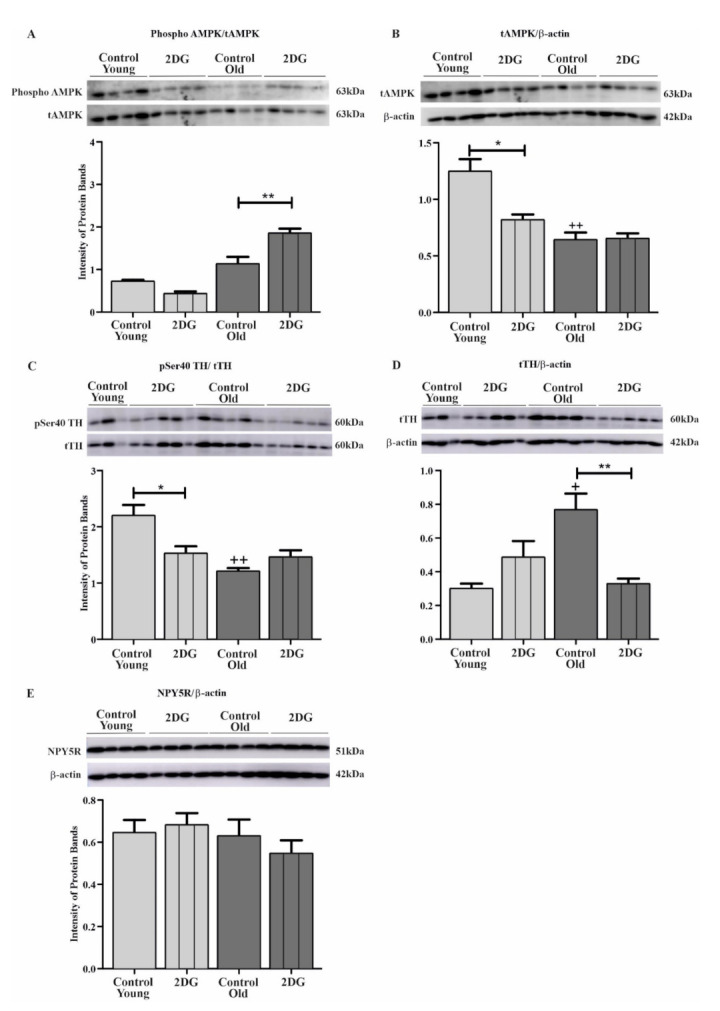
Effects of age and glucoprivation condition on the level of phosphorylated AMPK (**A**), total AMPK (tAMPK) (**B**), pSer40 TH (**C**), TH (**D**), and NPY5R (**E**). Each bar and associated error bar represents mean value of the intensity band ± SEM from one experiment (*n* = 6 per group). Representative Western blots (**A**–**D**) depicting effects of age and 2DG or saline; each lane represents a single animal. ++ (*p* < 0.01) and + (*p* < 0.05) represent significant differences between control groups of young and old rats, respectively, and ** (*p* < 0.01) and * (*p* < 0.05) represent significant differences between 2DG and control groups of the same-aged rats.

Figure 3 depicts the effect of glucoprivation on pSer40 TH, total TH (tTH), phosphorylated AMPK, total AMPK (tAMPK), and NPY5R in the CVLM across age groups. No significant changes in the level of phosphorylated AMPK and NPY5R were observed among the old control group compared with the younger control group (Figure 3A,E). Both tAMPK and pSer40 TH were lower in the old control group than in the young control group (*p* < 0.01) (Figure 3B,C). However, higher levels of the tTH protein were found in older rats relative to younger rats (*p* < 0.05) (Figure 3D).

In terms of phosphorylated AMPK, the young treatment group showed no changes, but an increase was observed in the old treatment group in comparison with its respective control group (*p* < 0.01) (Figure 3A). A decrease in tAMPK (*p* < 0.05) in response to glucoprivation was observed in the younger group, while 2DG failed to stimulate changes in tAMPK expression in the older group (Figure 3B). A significant decline in levels of pSer40 TH was observed in the 2DG-treated younger group compared with its control group (*p* < 0.05) (Figure 3C). Same-site TH phosphorylation remained unchanged in the older 2DG group compared with its control group (Figure 3C). Glucoprivation did not affect the expression of tTH protein in the young treatment group compared with the young control group (Figure 3D). However, a decrease in tTH expression (*p* < 0.01) was reported among 2DG-treated older rats (Figure 3D). No significant changes were reported for NPY5R in response to glucoprivation in the young and old groups relative to their controls (Figure 3E).

## 7. Discussion

This study revealed that the older group had higher levels of basal TH enzyme in the CVLM than the younger group. The adrenal gland, also a site for epinephrine production, showed similar increases in basal TH mRNA, protein, and activity [19,20,21]. Levels of circulating epinephrine appeared to be consistent among the old and young subjects despite the involvement of catecholamine synthesis [19,22,23]. Higher expression of TH protein in the CVLM of older rats could be an attribute that enables TH enzymes to be readily activated in order to meet higher catecholamine demands in times of stress. This study focused on pSer40 TH, which is site-phosphorylated by PKA, to which it was found that levels of pSer40 TH were lower among the older group than the younger group. Moreover, TH enzyme deactivation in the substantia nigra (SN) was said to increase with increasing levels of carbonyl content in the SN [24]. Interestingly, though PKA is not age-dependent, its regulator, adenylyl cyclase, is supposedly affected by age [25]. Therefore, attenuated cAMP/PKA signaling is probably responsible for the lower pSer40 TH level in older rats.

This study also found that NPY-Y5-receptor-like immunoreactivity (NPY5R-LI) in the CVLM increased with aging. A significant decrease in NPY1R, a separate NPY receptor subtype, was found in the hypothalamus [26]. Contrarily, the ventrolateral medulla (VLM) showed consistent NPY-like immunoreactivity (NPY-LI) in both young and old rats [27,28]. Injection of NPY into the CVLM produced a significant drop in blood pressure and heart rate [29]. As such, increasing NPY5R-LI in the CVLM may be counter-regulatory to age-associated increases in blood pressure [30,31,32]. Findings from this study revealed that the older group had less AMPK and a consistent AMPK-phosphorylation rate relative to the younger group. Several studies reported similar findings [33,34,35], though some showed increased AMPK phosphorylation and activity as age increased [36,37]. The function of AMPK is to promote ATP-generating processes and inhibit ATP-consuming processes [38]. Phosphorylation of acetyl-CoA carboxylase (ACC) decreases with age [33,37]. Therefore, lower AMPK activity could contribute to age-related dysregulation of fat metabolism [39,40]. Inhibition of AMPK signaling increases neurogenesis in the hippocampus, suggesting that the upregulation of fatty-acid synthesis is due to decreased AMPK signaling, which promotes cell proliferation [41]. This finding also explains the reason for the high AMPK-like immunoreactivity (AMPK-LI) among the older group.

An investigation on the effect of 2DG-induced glucoprivation on CVLM C1 neurons among young and old groups was conducted. A significant decrease in pSer40 TH levels post-2DG injection was found within the young group but not the older group. Other studies, however, observed different trends among different phosphorylation sites (Ser19, Ser31 TH) during glucoprivation [13,42], although 2DG is said to increase activation of CVLM neurons [43]. A 2DG-induced increase in epinephrine could exert negative feedback towards TH phosphorylation, controlling levels of catecholamine secretion, thus resulting in the lower pSer40 TH found in the young treatment group. However, a similar decrease was not observed in the old treatment group, suggesting an aging-related absence of 2DG-induced TH activation. An increase in age also diminished the TH response towards stressors, such as long-term exercise and reserpine administration [20,44]. The TH protein was not altered by 2DG in the younger group, but a decline in TH was observed among the older group. Damanhuri et al. also reported similar findings based on a study administering 2DG to young rats [13]. Nonetheless, regulation of TH in CVLM neurons under glucoprivation appeared to be age-specific, where phosphorylation was more likely among the younger group, whereas TH in the older group was regulated by protein expression. However, the mechanism explaining the age difference remains unclear.

Insulin and 2DG are said to significantly increase the activation and activity of AMPK in the hypothalamus, specifically in the PVN, ARC, and VMH [45,46]. However, this study found no significant changes in the phosphorylation of AMPK within CVLM neurons in the young treatment group. High levels of glucose in the bloodstream could trigger glucagon to activate insulin secretion, promoting cell glucose uptake [47,48,49,50]. Thus, reduced AMPK protein within CVLM neurons could be due to the inhibitory action of insulin [51]. Unlike the younger group, the older group showed higher phosphorylation of AMPK. However, similar levels of AMPK protein were observed in response to 2DG-induced glucoprivation among both age groups. Furthermore, 2DG caused a decrease in cellular ATP levels [52], resulting in higher AMPK activation. Glucose availability can be increased through the role of AMPK, which promotes ATP-producing processes and inhibits ATP-consuming processes [38]. Overall, these findings suggest that the older group’s counter-regulatory responses were activated via AMPK signaling.

**Figure 4:**
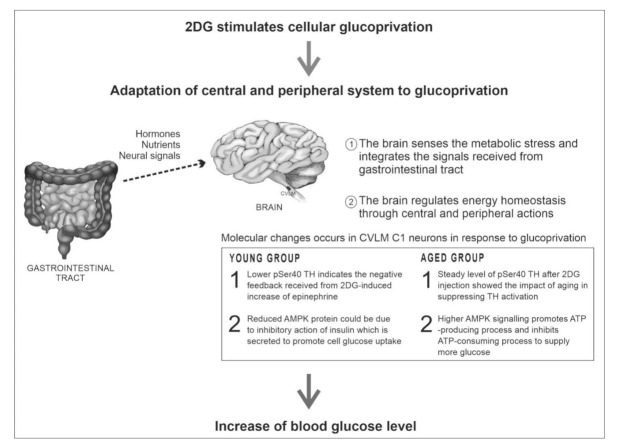
Summary of the glucose counterregulatory responses in CVLM neurons following glucoprivation in young and aged rats [56].

Administration of 2DG to both age groups revealed no significant changes towards NPY5R expression in CVLM neurons, therefore highlighting the need for further studies on the role of glucoprivation in the activation of NPY5R. From a previous study, the use of NPY5R antagonists revealed the role of NPY5R in synaptic strength and epinephrine secretion during food deprivation [53]. The NPY5R ligand, NPY, has been extensively studied, enforcing its participation in the activation of counter-regulatory responses [53,54,55]. In conclusion, protein modifications observed in the CVLM play a role in the feeding pathway as aging takes place. Aging reduces TH activation in the CVLM, thus affecting intended adrenergic signaling. There might be an association between increased NPY-like immunoreactivity and reduced AMPK activity among the older group with age-induced secondary changes (e.g., high blood pressure and dysregulated fat metabolism). Administering 2DG resulted in significant increases in blood glucose among both age groups, signifying the activation of counter-regulatory responses (CRR) towards glucoprivation. The involvement of CVLM C1 neurons in CRR is further backed by reduced pSer40 TH and AMPK proteins within the young group. Although aging suppressed TH activation in CVLM C1 neurons in response to 2DG, the older group was able to activate CRR by increasing AMPK signaling in CVLM C1 neurons. These findings suggest the role of CVLM C1 neurons in the counter-regulatory responses of young and old rats (see Figure 4). Altering protein changes in aging CVLM C1 neurons may attenuate responses to glucoprivation, thus explaining the decline in food intake among the elderly.

## Data Availability

Not applicable.

## Supplementary Materials

The following are available online at https://www.mdpi.com/article/10.3390/biom12030449/s1, Figure S1: Representative blots of AMPK (A), TH (B), and NPY5R (C).
