# Supplementary figures and images for: Attenuation of the Counter-Regulatory Glucose Response in CVLM C1 Neurons: A Possible Explanation for Anorexia of Aging
Source: Biomolecules. 2022 Mar 14;12(3):449. doi: 10.3390/biom12030449 (PMC8945993; doi:10.3390/biom12030449)

**A**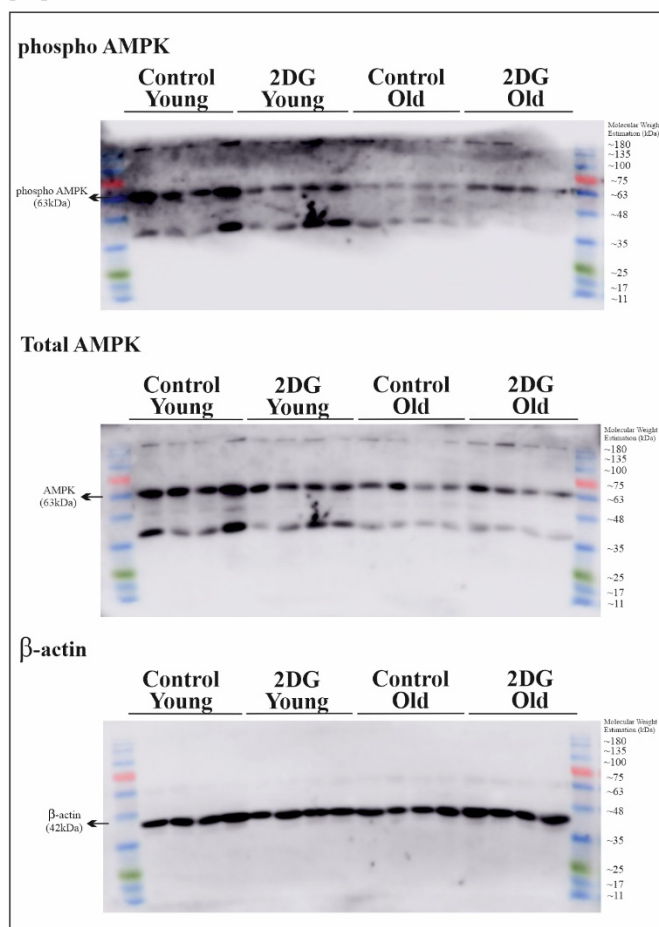**B**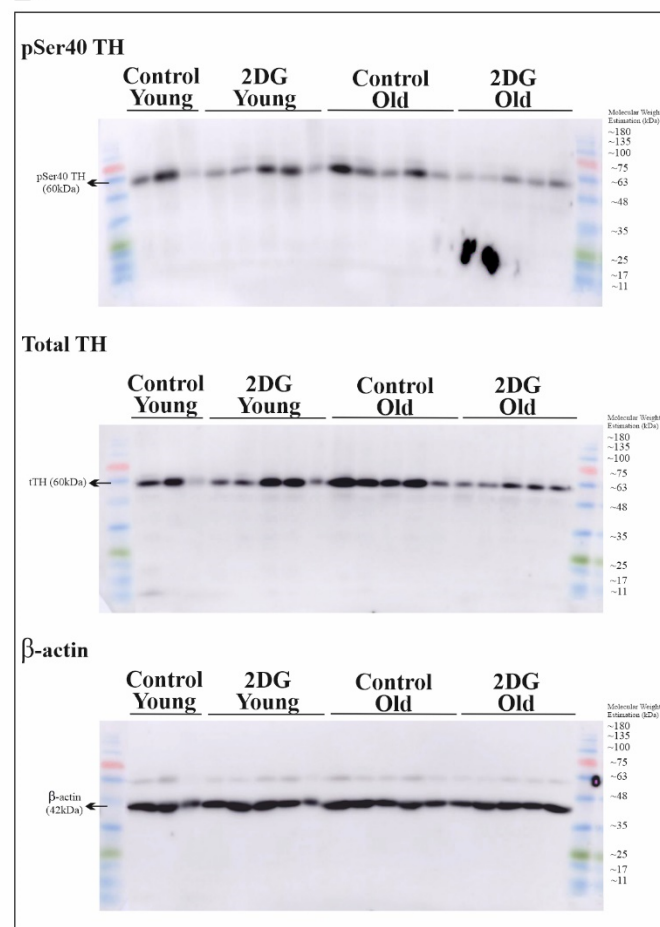**C**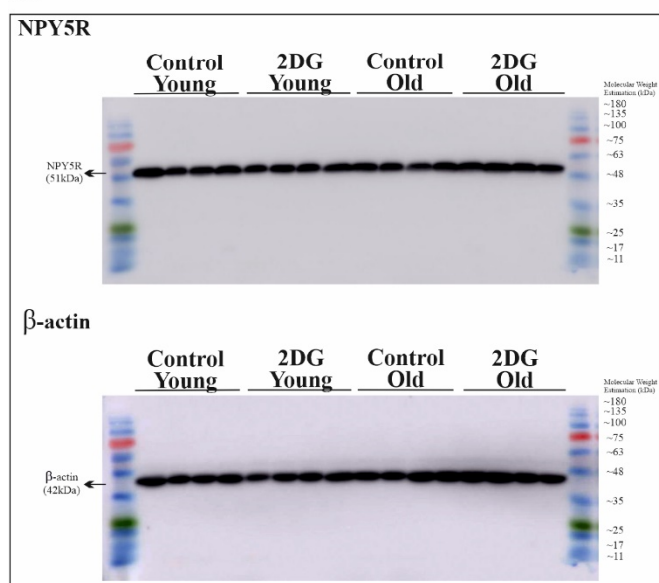

**Figure S1.** Representative blots of AMPK (A), TH (B), and NPY5R (C).

Supplement: Supplementary file 1 [file biomolecules-12-00449-s001.zip › biomolecules-1467006-supplementary.pdf]
